# Effect of polyunsaturated fatty acids on drug-sensitive and resistant tumor cells *in vitro*

**DOI:** 10.1186/1476-511X-10-159

**Published:** 2011-09-14

**Authors:** Undurti N Das, N Madhavi

**Affiliations:** 1 Jawaharlal Nehru Technological University, Kakinada-533 003, India; 2 UND Life Sciences, 13800 Fairhill Road, #321, Shaker Heights, OH 44120, USA; 3 Bio-Science Research Centre, Gayatri Vidya Parishad College of Engineering, Visakhapatnam-530 048, India

**Keywords:** Polyunsaturated fatty acids, essential fatty acids, free radicals, vincristine, lipid peroxidation, cancer, uptake, efflux, arachidonic acid, eicosapentaenoic acid, docosahexaenoic acid, gamma-linolenic acid, linoleic acid, linolenic acid

## Abstract

Previous studies showed that γ-linolenic acid (GLA, 18: 3 ω-6), arachidonic acid (AA, 20:4 ω -6), eicosapentaenoic acid (EPA, 20: 5 ω -3) and docosahexaenoic acid (DHA, 22:6 ω -3) have selective tumoricidal action. In the present study, it was observed that dihomo-gamma-linolenic acid (DGLA) and AA, EPA and DHA have cytotoxic action on both vincristine-sensitive (KB-3-1) and resistant (KB-Ch^R^-8-5) cancer cells *in vitro *that appeared to be a free-radical dependent process but not due to the formation of prostaglandins, leukotrienes and thromboxanes. Uptake of vincristine and fatty acids was higher while their efflux was lower in KB-3-1 cells compared with KB-Ch^R^-8-5 cells, suggesting that drug resistant cells have an effective efflux pump. GLA, DGLA, AA, EPA and DHA enhanced the uptake and decreased efflux in both drug-sensitive and drug-resistant cells and augmented the susceptibility of tumor cells especially, of drug-resistant cells to the cytotoxic action of vincristine. These results suggest that certain polyunsaturated fatty acids have tumoricidal action and are capable of enhancing the cytotoxic action of anti-cancer drugs specifically, on drug-resistant cells by enhancing drug uptake and reducing its efflux. Thus, polyunsaturated fatty acids either by themselves or in combination with chemotherapeutic drugs have the potential as anti-cancer molecules.

## Introduction

It is desirable to kill tumor cells selectively without harming normal cells. But, currently available drugs and radiation fail to kill only tumor cells and cause significant side effects that are undesirable. Anti-VEGF (vascular endothelial growth factor) and anti-EGF (epidermal growth factor) and other monoclonal antibodies developed for use in cancer do possess some degree of specific action on tumor cells yet are not very effective. In view of this, further studies are needed to identify newer molecules that possess selective tumoricidal property that are less toxic but have predictable actions.

Previously, we and others showed that some polyunsaturated fatty acids (PUFAs) induced apoptosis of tumor cells with little or no cytotoxic action on normal cells under the conditions employed [1-10]. It was observed that of all the fatty acids tested, GLA was the most effective in selectively killing the tumor cells. In a co-culture experiment wherein normal human skin fibroblasts (CCD-41-SK) and human breast cancer cells (ZR-75-1) were grown together in a petri dish and supplemented with GLA, only human breast cancer cells were eliminated without any effect on normal skin fibroblasts [11]. These results reconfirmed that GLA and possibly, other PUFAs under some specific conditions show selective tumoricidal action at least *in vitro*. GLA and other unsaturated other fatty acids induced apoptosis of tumor cells by enhancing the release of cytochrome *c*, activating caspase-3, suppressing Akt phosphorylation and modulating p38 MAPK in the phosphorylation of p53 at Ser15, a site which is associated with DNA damage (9, 10). These molecular changes were found to be significantly associated with enhanced degree of lipid peroxidation in the fatty acid supplemented tumor cells (1-5, 9). GLA and other PUFAs were also found to be capable of suppressing the expression of oncogenes *ras *and *Bcl-2 *and enhance p53 activity and thus, induce apoptosis of tumor cells [12].

In an extension of these studies, it was noted that cyclo-oxygenase (CO) and lipoxygenase (LO) inhibitors blocked the tumoricidal action of GLA on human cervical carcinoma, HeLa cells; whereas anti-oxidants inhibited cytotoxic action of GLA on human breast cancer, ZR-75-1, cells [1,2,4]. Prostaglandins (PGE_1_, PGE_2_, PGF_2α_, PGI_2_) and LO products of GLA: 13-HPODE and 6-HPODE, inhibited the growth of HeLa cells [2,4]. LO products were more potent than PGs in inhibiting of HeLa cell growth [4] that was confirmed by the observation that a 9-fold increased formation of hydroxides occurred in HeLa cells treated with GLA. These results suggest that both CO and LO products and free radicals are involved in the tumoricidal action of GLA. A significant increase in the formation of free radicals and lipid peroxides was noted only in tumor cells treated with GLA (GLA > AA > EPA > LA) compared to untreated tumor cells or GLA-treated normal skin fibroblasts [1,4,5,9,13,14], suggesting that the involvement of CO and LO products, free radicals and lipid peroxides in the tumoricidal action of GLA and PUFAs varies depending on the cell type that is being tested.

Drug resistance is a major issue in the management of cancer. Hence, methods or strategies to prevent and/or reverse tumor cell dug resistance are needed. Previously, we observed that GLA could kill even drug resistant tumor cells *in vitro *[15]. GLA augmented the cytotoxic action of anti-cancer drugs cis-platinum and doxorubicin [16]. Studies by Menendez *et al *[17], Hernandez *et al *[18], and Rudra *et al *[19] confirmed that GLA and other unsaturated fatty acids augment tumoricidal actions of anti-cancer drugs and a synergism exists between conventional anti-cancer drugs and GLA. But, it is not clear as to the exact mechanism by which this synergism between anti-cancer drugs and fatty acids occurs. In the present study, we studied the effects of various PUFAs on drug-sensitive and drug-resistant tumor cells, possible potentiation of the tumoricidal action of sub-optimal anti-cancer drugs on drug-resistant cells and possible mechanisms(s) involved in these actions.

## Materials and methods

### Cells and culture conditions

Human cervical carcinoma cells which are sensitive (KB-3-1) and resistant (KB-Ch^R^-8-5) to the cytotoxic action of vincristine respectively were used for this study. KB-3-1 and KB-Ch^R^-8-5 are HeLa variant cell lines.

These cells were grown and maintained in NUNC culture flasks in bicarbonate buffered DMEM with 10% fetal calf serum and L-glutamine at 37°C in a 5% CO_2 _humidified incubator. KB-Ch^R^-8-5 cells, which are 4-fold resistant to colchicine, were grown in the continuous presence of colchicine [15,20-22]. Cells were seeded at 1 × 10^4 ^cells/ml/well in 24 well tissue culture plates for various studies. One day after seeding, the medium was removed and fresh medium with/without various fatty acids, and other compound solutions was added depending on the experimental protocol. The fatty acids were initially dissolved in 95% ethanol and the final concentration of ethanol was not more than 0.02% in all control and fatty acid supplemented cultures.

### Cell viability studies

KB-3-1 and KB-Ch^R^-8-5 cells treated with various fatty acids and other compounds were also assessed for their viability at the end of various incubation periods. The viability of cells was assessed by using Trypan blue dye exclusion method.

### Thymidine incorporation studies

To study the growth of KB-3-1 and KB-Ch^R^-8-5 cells in the presence of various concentrations of different fatty acids and the effect of cyclo-oxygenase (CO) and lipoxygenase (LO) inhibitors, anti-oxidants and calmodulin antagonists, the ability of cells to incorporate radiolabeled thymidine as a function of DNA synthesis was used. One day after seeding, 0.5 μCi of labeled thymidine (specific activity 18, 500 mCi/mmol) was added 6 hours before harvesting the cells. At the end of the incubation period, the cells were washed at least three times with PBS (pH 7.4), detached by trypsinization, extracted for DNA, and counted in a liquid scintillation counter on days 1, 2 and 3 to assess cell growth.

### NBT reduction

The superoxide anion (O_2_**^.-^**) can reduce nitroblue tetrazolium (NBT) ion to the insoluble formazan. This is a simple, reliable and acceptable method of assaying superoxide anion and possibly, other free radicals [1,18-21]. KB-3-1 and KB-Ch^R^-8-5 cells grown with or without fatty acids, with or without other compounds for 24, 48 a 72 hours were checked for their ability to reduce NBT by incubation with 0.1% NBT dissolved in phosphate buffered saline (pH 7.4) for 2 hours at the end of each time period. Termination of the assay (final assay volume 0.3 ml) was done by adding 0.6 ml of glacial acetic acid into which the reduced NBT dye was extracted and read at 560 nm as described previously [1,23-25].

### Hydrogen peroxide formation

The amount of H_2_O_2 _formed in KB-3-1 and KB-Ch^R^-8-5 cells with and without fatty acid treatment and other compounds was estimated by the horse-radish peroxidase method [26-28].

### Lipid peroxidation

The total amount of lipid peroxidation products formed in the cells was estimated using the thiobarbituric acid (TBA) method [29,30].

### Uptake of radiolabeled vincristine

The uptake of radiolabeled vincristine was studied using ^3^H-vincristine sulfate (specific activity 6.2 Ci mmol^-1^). To 1 × 10^4 ^cells/ml/well, one day after seeding, the medium was replaced with fresh medium along with 50 nm [^3^H] vincristine was added and incubated for further tile periods. At the end of 1, 2, 4, 6 and 12 hours of addition of vincristine, the cells were washed, detached by trypsinization and counted in a liquid scintillation counter. To study the effect of fatty acids on vincristine uptake, one day after seeding KB-3-1 and KB-Ch^R^-8-5 cells were incubated with different doses of fatty acids ranging from 10 to 40 μg/ml. After 6 hours of addition of fatty acids, 50 nm of [^3^H] vincristine was added and incubated for further time periods. At the end of 1, 2 and 4 hours of radiolabeled vincristine addition; cells were washed thrice with PBS, detached by trypsinization and counted in a liquid scintillation counter.

All the experiments were performed in quadruplicate and repeated at least twice. Result are expressed as Mean ± SD and analyzed using Student's t test and/or one-was analysis of variance followed by Tukey's Honesty Significant Difference (HSD) test.

### Efflux of [^3^H] vincristine in the presence of fatty acids

1 × 10^4 ^cells/ml/well were seeded in 24-well plates. Cells were allowed to attach to plastic overnight and at the end of 24 hours, medium was aspirated and fresh medium added along with fatty acids whose concentrations ranged from 5-40 μg/ml. At the end of 4 hours of fatty acid incubation, 50 nM [^3^H] vincristine was added and incubated for another 2 hours after which the medium was discarded and cells were washed with PBS. 0.5 ml of phenol red free DMEM was added to the cells and incubated for 1, 2 and 4 hours. At the end of the incubation period, the medium was aspirated and counted in a liquid scintillation counter.

### Uptake of radiolabeled fatty acids

The uptake of radiolabeled fatty acids (ALA, AA and EPA) was studied using ^14^C-labeled ALA, AA and EPA (specific activity 54, 55, 59 mCi/mmol). To 1 × 10^4 ^cells/ml/well, one day after seeding, the medium was replaced with fresh medium along with 50 nm [^14^C] of respective fatty acid was added and incubated for further tile periods. At the end of 6, 12, 24, 28, and 72 hours of addition of fatty acid, the cells were washed, detached by trypsinization and counted in a liquid scintillation counter.

### Efflux of [^14^C] fatty acids by vincristine-sensitive (KB-3-1) and vincristine-resistant (KB-Ch^R^-8-5) cells

1 × 10^4 ^cells/ml/well were seeded in 24-well plates. Cells were allowed to attach to plastic overnight and at the end of 24 hours, medium was aspirated and fresh medium added along with labeled fatty acids. At the end of 6, 12, 24, 48, and 72 hours4 hours of labelled fatty acid incubation, the medium was aspirated and counted in a liquid scintillation counter.

### Statistics

All the experiments were performed in quadruplicate and repeated at least twice. Result are expressed as Mean ± SD and analyzed using Student's "t" test and/or one-was analysis of variance followed by Tukey's Honesty Significant Difference (HSD) test depending on the experimental protocols.

## Results

### Effect of various fatty acids on the survival of vincristine-sensitive and resistant tumor cells *in vitro*

**Figure 1:**
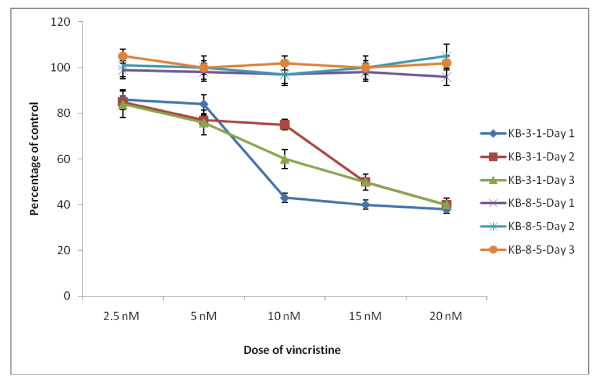
**Effect of various concentrations of vincristine on the survival of vincristine sensitive (KB-3-1) and resistant (KB-Ch^R^-8-5) tumor cells *in vitro *expressed as percentage of control**. All values are Mean ± SD.

Results shown in Figure 1 clearly indicate that KB-Ch^R^-8-5 cells are resistant to the cytotoxic action of vincristine whereas KB-3-1 cells are more sensitive to its action.

**Figure 2:**
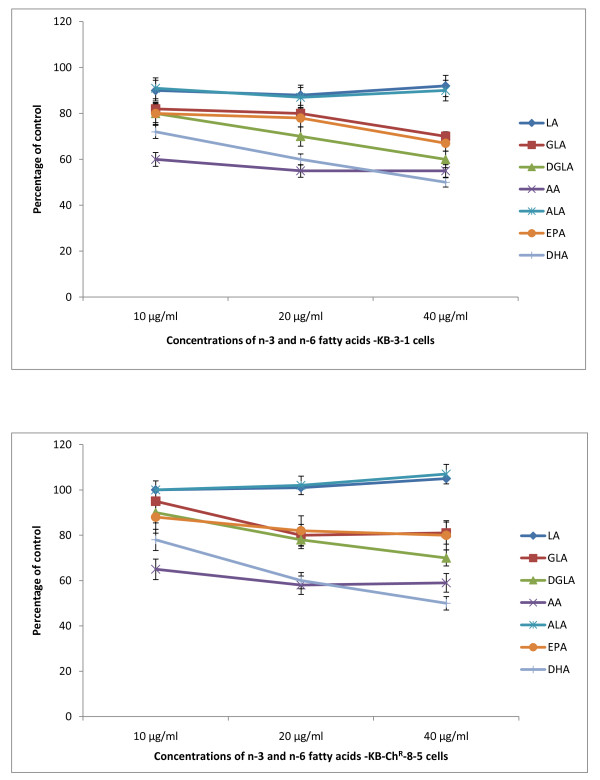
**Effect of different doses of n-6 and n-3 fatty acids on the survival of vincristine-sensitive (KB-3-1) and vincristine-resistant (K-B-Ch^R^-8-5) tumor cells *in vitro *on day 3 expressed as percentage of control**. All values are Mean ± SD.

**Figure 3:**
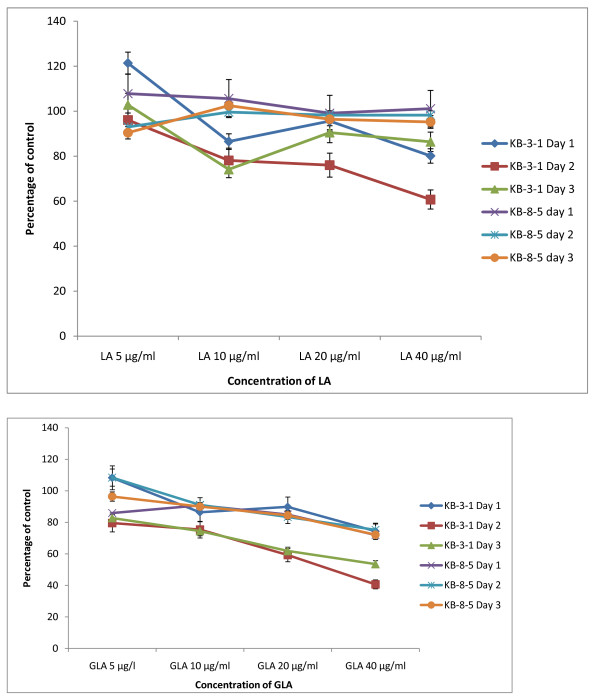
**Effect of different doses of LA and GLA on thymidine uptake by vincristine-sensitive (KB-3-1) and vincristine-resistant (K-B-Ch^R^-8-5) tumor cells *in vitro *on days 1-3 expressed as percentage of control**. All values are Mean ± SD.

**Figure 4:**
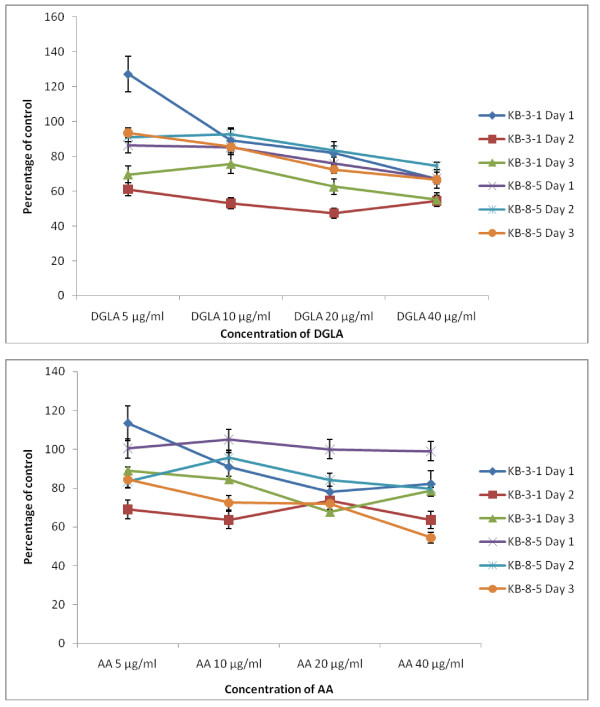
**Effect of different doses of DGLA and AA on thymidine uptake by vincristine-sensitive (KB-3-1) and vincristine-resistant (K-B-Ch^R^-8-5) tumor cells *in vitro *on days 1-3 expressed as percentage of control**. All values are Mean ± SD.

**Figure 5:**
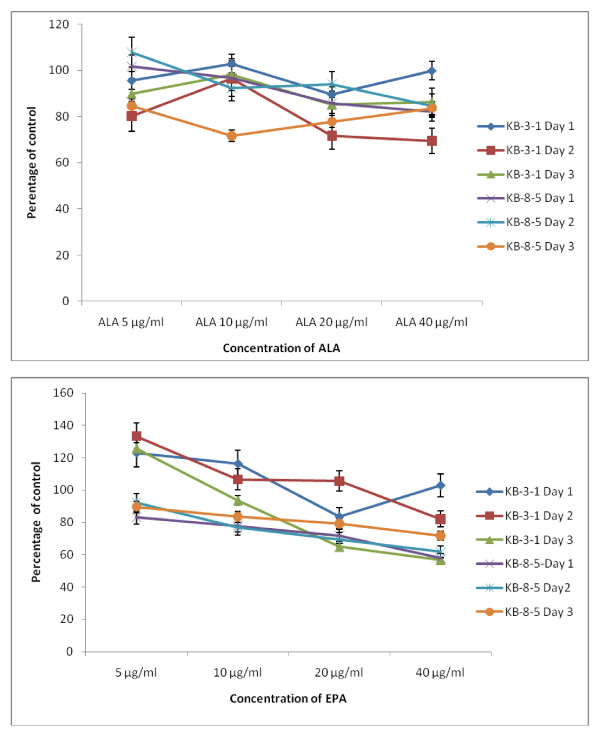
**Effect of different doses of ALA and EPA on thymidine uptake by vincristine-sensitive (KB-3-1) and vincristine-resistant (K-B-Ch^R^-8-5) tumor cells *in vitro *on days 1-3 expressed as percentage of control**. All values are Mean ± SD.

**Figure 6:**
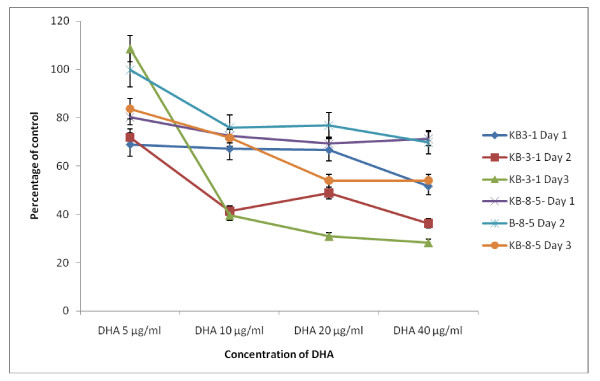
**Effect of different doses of DHA on thymidine uptake by vincristine-sensitive (KB-3-1) and vincristine-resistant (K-B-Ch^R^-8-5) tumor cells *in vitro *on days 1-3 expressed as percentage of control**. All values are Mean ± SD.

The data in Figure 2 shows that DHA, EPA and AA are the most effective of all the fatty acids tested with regard to their cytostatic/toxic effects on KB-3- and KB-ChR-8-5 cells. The cytostatic/cytotoxic effect of various fatty acids tested is further evident from the thymidine incorporation studies given in Figures 3, 4, 5 and 6. These results showed that thymidine incorporation decreases as the dose of fatty acids is increased. The cytostatic/cytotoxic action of fatty acids on KB-3-1 cells in order of potency is as follows: DHA > EPA > GLA = DGLA > AA > LA > ALA. On the other hand, the cytostatic/cytotoxic action of fatty acids on KB-Ch^R^-8-5, the vincristine-resistant cells, in order of potency is as follows: DHA > AA > DGLA = EPA = GLA > LA > ALA. These results indicate that both vincristine-sensitive and resistant cells are almost equal in their sensitivity to the cytotoxic action of various fatty acids tested.

### Effect of CO, LO inhibitors, anti-oxidants and calmodulin antagonists on the cytotoxic action of polyunsaturated fatty acids

**Figure 7:**
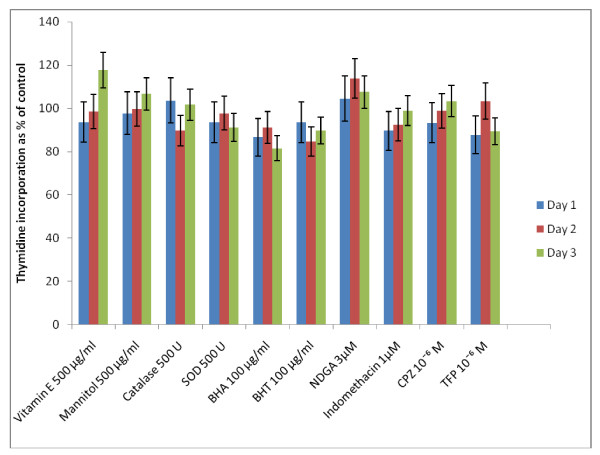
**Effect of various anti-oxidants, cyclo-oxygenase and lipoxygenase inhibitors and calmodulin antagonists on thymidine incorporation in KB-3-1 cells in vitro on day 1, 2 and 3**. All values are Mean ± SD.

**Figure 8:**
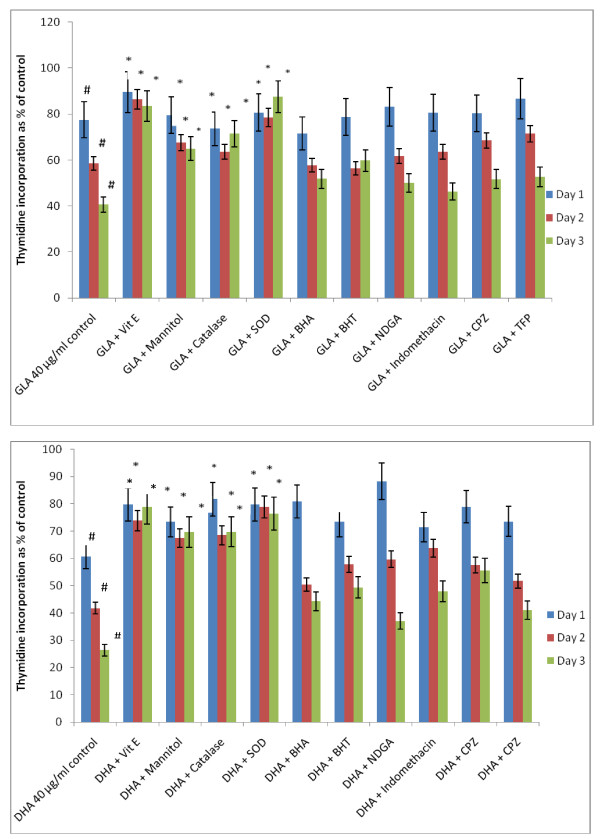
**Effect of various anti-oxidants, cyclo-oxygenase and lipoxygenase inhibitors and calmodulin antagonists on GLA and DHA-induced growth inhibition of KB-3-1 cells in vitro**.

Since polyunsaturated fatty acids (PUFAs) form precursors to various prostaglandins (PGs), leukotrienes (LTs) and thromboxanes (TXs), induce the generation of free radicals and modulate the action of calmodulins, the possibility that the fatty acids tested are able to bring about their cytotoxic action on KB-3-1 cells based on these mechanisms was tested using CO and LO inhibitors, various anti-oxidants and calmodulin antagonists. The results of this study done with GLA and DHA as representative of n-6 and n-3 fatty acids given in Figures 7 and 8 revealed that indomethacin, a CO inhibitor, and NDGA, a LO inhibitor, were ineffective in blocking the cytotoxic action of GLA and DHA.

It is evident from the results shown in Figure 8 that both indomethacin and NDGA, a CO and LO inhibitors respectively, were ineffective in blocking the cytotoxic action of GLA on KB-3-1 cells in vitro. Both vitamin E and SOD completely blocked the cytotoxic action of GLA on KB-3-1 cells, while synthetic anti-oxidants such as BHA and BHT and calmodulin antagonists: chlorpromazine (CPZ) and trifluoperazine (TFP) were ineffective. On the other hand, both mannitol and catalase were effective in inhibiting the GLA-induced cytotoxicity only by about 40-60%. These results are more evident by day 3.

Similar to the results seen with GLA, even with DHA it is clear from the results shown in Figure 8 that both indomethacin and NDGA, a CO and LO inhibitors respectively, were ineffective in blocking the cytotoxic action of DHA on KB-3-1 cells in vitro. Both vitamin E and SOD completely blocked the cytotoxic action of GLA on KB-3-1 cells, while synthetic anti-oxidants such as BHA and BHT and calmodulin antagonists: chlorpromazine (CPZ) and trifluoperazine (TFP) were ineffective. On the other hand, unlike the results seen with GLA, both mannitol and catalase were as effective as that of vitamin E and SOD in inhibiting the DHA-induced cytotoxicity. These results are more evident on day 3.

### Effect of PUFAs on free radical generation by KB-3-1 and KB-Ch^R^-8-5 cells *in vitro*

**Figure 9:**
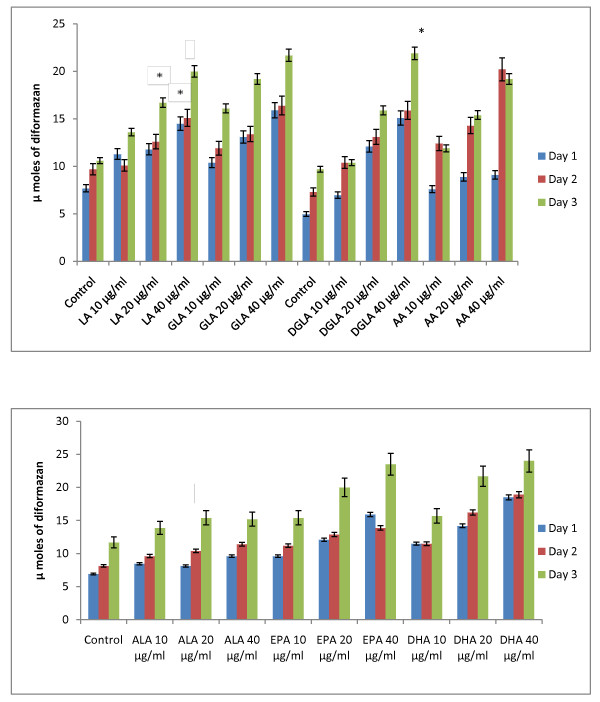
**Effect of various n-6 and n-3 fatty acids on the amount of superoxide anion generated in KB-3-1 cells measured as NBT reduction and expressed as μ moles of diformazan formed in vitro**. All values are Mean ± SD. * P < 0.05 compared to control.

**Figure 10:**
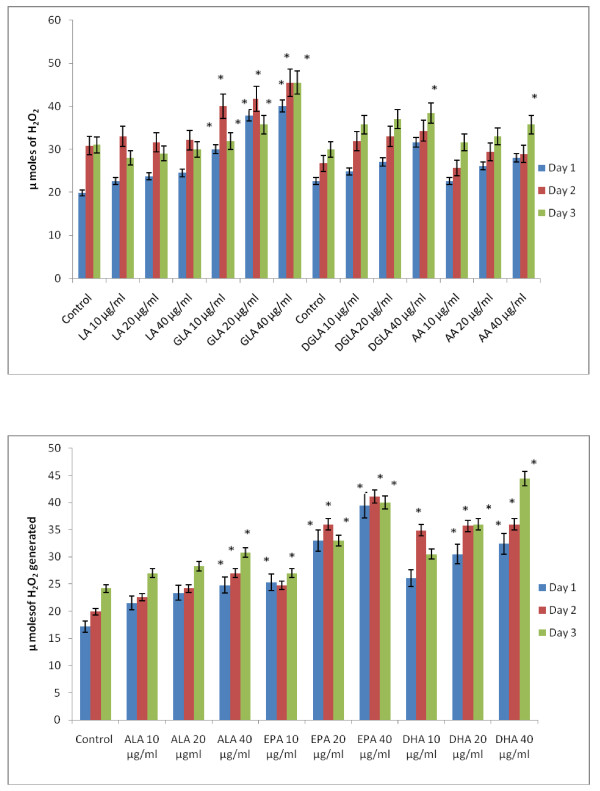
**Effect of various concentrations of different n-6 and n-3 fatty acids on the generation of H_2_O_2 _by KB-3-1 cells *in vitro***. All values are expressed as μ moles of H_2_O_2 _formed. All values are Mean ± SD. * P < 0.05 compared to control.

**Figure 11:**
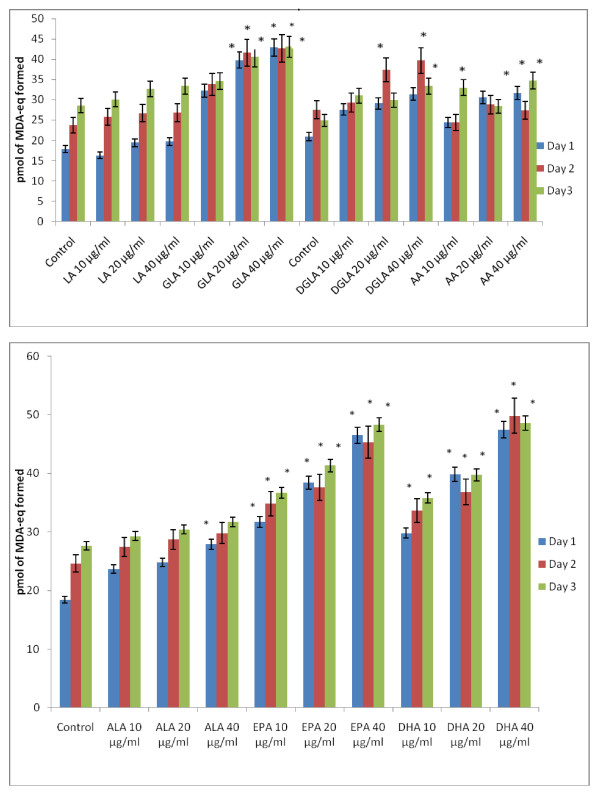
**Effect of different doses of n-6 and n-3 fatty acids on the formation of lipid peroxides (expressed as pmoles of MDA-eq formed) in KB-3-1 cells in vitro**. All values are Mean ± SD. *P < 0.05 compared to control.

**Figure 12:**
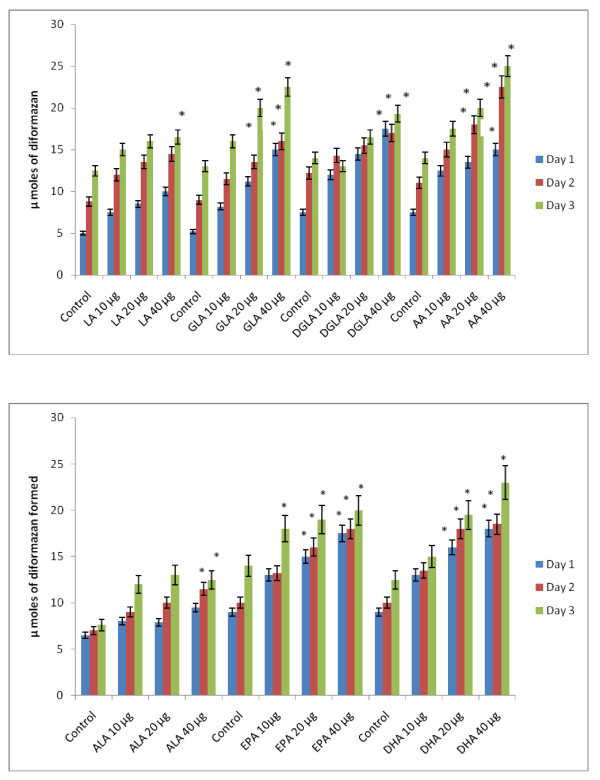
**Effect of various n-6 and n-3 fatty acids on the amount of superoxide anion generated in KB-Ch^R^-8-5 measured as NBT reduction and expressed as μ moles of diformazan formed in vitro**. All values are Mean ± SD. * P < 0.05 compared to control.

**Figure 13:**
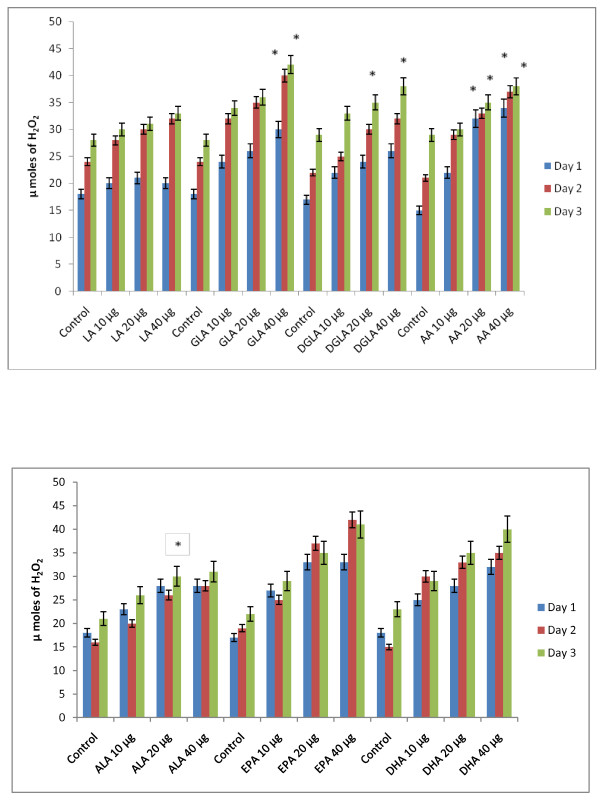
**Effect of various n-6 and n-3 fatty acids on the amount of hydrogen peroxide generated in KB-Ch^R^-8-5 in vitro**. All values are Mean ± SD. * P < 0.05 compared to control.

**Figure 14:**
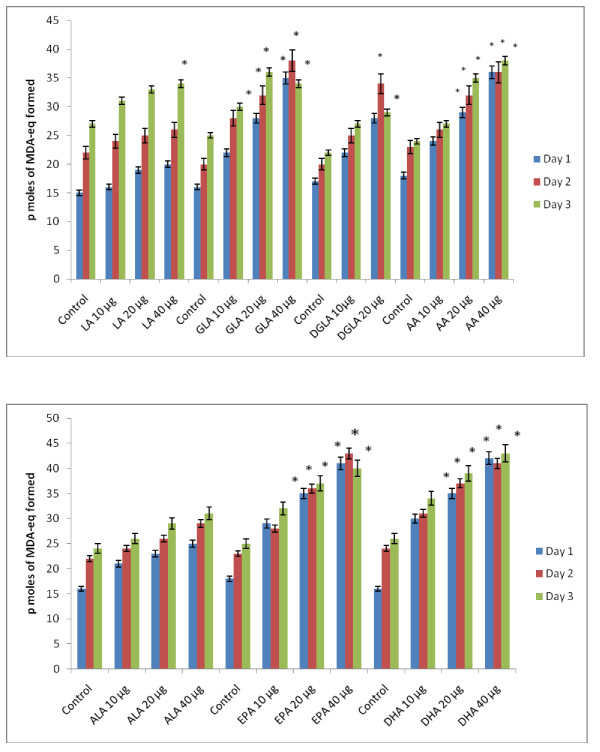
**Effect of different doses of n-6 and n-3 fatty acids on the formation of lipid peroxides (expressed as pmoles of MDA-eq formed) in KB-Ch^R^-8-5 cells in vitro**. All values are Mean ± SD. * P < 0.05 compared to control.

Since vitamin E and SOD (superoxide dismutase), an effective anti-oxidant and a superoxide quencher respectively, blocked the cytotoxic action of fatty acids on KB-3-1 and KB-Ch^R^-8-5 cells, and since free radical generation can be modulated by fatty acids, the effect of various n-6 and n-3 fatty acids on superoxide anion and H_2_O_2 _generation and the formation of lipid peroxides in these cells was studied. The results shown in Figures 9, 10, 11, 12, 13 and 14 indicate that although all the fatty acids are effective, relatively higher amounts of superoxide and H_2_O_2 _generation and formation of lipid peroxides were observed in GLA, AA, EPA and DHA-treated KB-3-1 and KB-Ch^R^-8-5 cells. It is noteworthy that enhanced generation of superoxide anion, H_2_O_2 _and lipid peroxides induced by GLA, AA, EPA and DHA corresponded to their cytotoxic action on KB-3-1 and KB- Ch^R^-8-5 cells.

**Figure 15:**
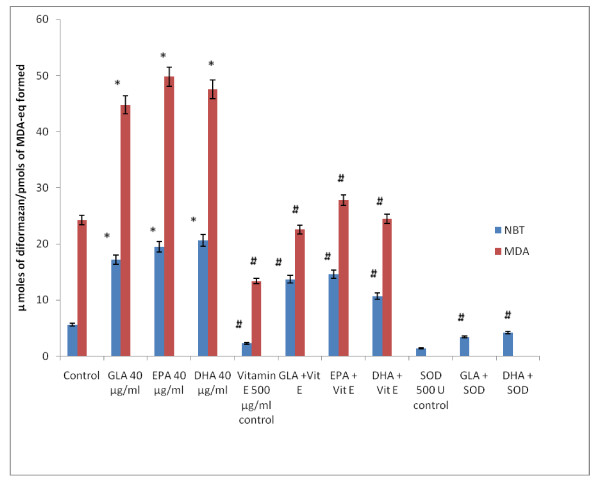
**Effect of anti-oxidants vitamin E and SOD (superoxide dismutase) on the generation of free radicals (superoxide anion) and lipid peroxides induced by GLA, EPA and DHA in KB-3-1 cells in vitro on day 3 of incubation with fatty acids**. Superoxide anion generated in KB-3-1 cells was measured as NBT reduction (expressed as μ moles of diformazan formed) and lipid peroxides formed is measured by TBA reaction and expressed as pmoles of MDA-eq formed. All studies were done on day 3 following incubation with 40 μg/ml of fatty acids with or without vitamin E or SOD. All values are Mean ± SD. * P < 0.05 compared to control; #P < 0.05 compared to fatty acid treatment.

It is interesting to note that NBT reduction reaction (that detects superoxide anion generation) induced by fatty acids could be completely blocked by SOD suggesting that under the conditions employed, what is being measured, in fact, is the superoxide anion radical. The involvement of free radicals and lipid peroxides in the fatty acid-induced cytotoxicity is supported by the observation that both free radical generation and the formation of lipid peroxidation products (measured as MDA-reactive material) in KB-3-1 cells can be completely inhibited by vitamin E and SOD. This experiment was performed with 40 μg/ml of GLA, EPA and DHA fatty acids in KB-3-1 cells and the results are given in Figure 15.

### Uptake and efflux of radiolabeled vincristine in KB-3-1 and KB-Ch^R^-8-5 cells *in vitro*

**Figure 16:**
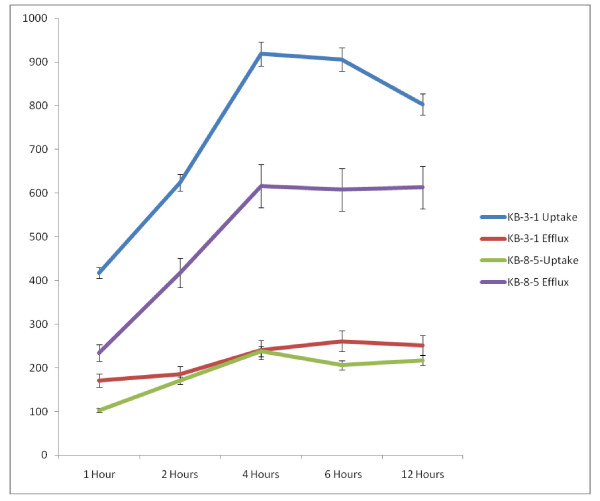
**Uptake and efflux of vincristine (radiolabeled) by vincristine-sensitive (KB-3-1) and vincristine-resistant (KB-Ch^R^-8-5) tumor cells *in vitro *at different time intervals**.

In order to understand the mechanism as to why KB-3-1 cells are sensitive whereas KB-Ch^R^-8-5 cells are resistant to the cytotoxic action of vincristine, the uptake and efflux of radiolabeled vincristine in these cells was studied. The results shown in Figure 16 revealed that the uptake of vincristine is almost 4 to 5-fold higher in KB-3-1 cells compared with the uptake by KB-Ch^R^-8-5 cells. On the other hand, the efflux of vincristine is 1 1/2 to 3 times less in KB-3-1 cells compared with the efflux shown by KB-Ch^R^-8-5 cells. This difference in the uptake and efflux ultimately resulted in 2-3 fold higher concentration of vincristine in KB-3-1 cells compared to KB-Ch^R^-8-5 cells. This higher intracellular concentration of vincristine in KB-3-1 cells could be responsible for their sensitivity to the cytotoxic action of vincristine, whereas KB-Ch^R^-8-5 cell are resistant to the cytotoxic action of vincristine since they are able to effectively reduce the intracellular concentration of vincristine by an effective efflux pump. Thus, the uptake of vincristine is high in KB-3-1 cells and the efflux of the drug is minimal while vincristine- resistant KB-Ch^R^-8-5 cells showed minimal uptake and very effective efflux mechanism to remove vincristine from within the cell. As a result, the accumulation of vincristine is not seen in resistant cells KB-Ch^R^-8-5 cells.

Since, cell membrane properties determine the uptake and efflux of drugs, it is expected that supplementation of PUFAs that get incorporated into the cell membrane lipids to vincristine-resistant cells may render them sensitive to the cytotoxic action of vincristine by enhancing its uptake and reducing the efflux. Studies performed to verify this possibility showed that this is indeed the case as presented below.

### Uptake and efflux of [^3^H] vincristine by KB-3-1 and KB-Ch^R^-8-5 cells in the presence of various fatty acids

#### Effect of various n-6 fatty acids

##### Studies with LA

**Figure 17:**
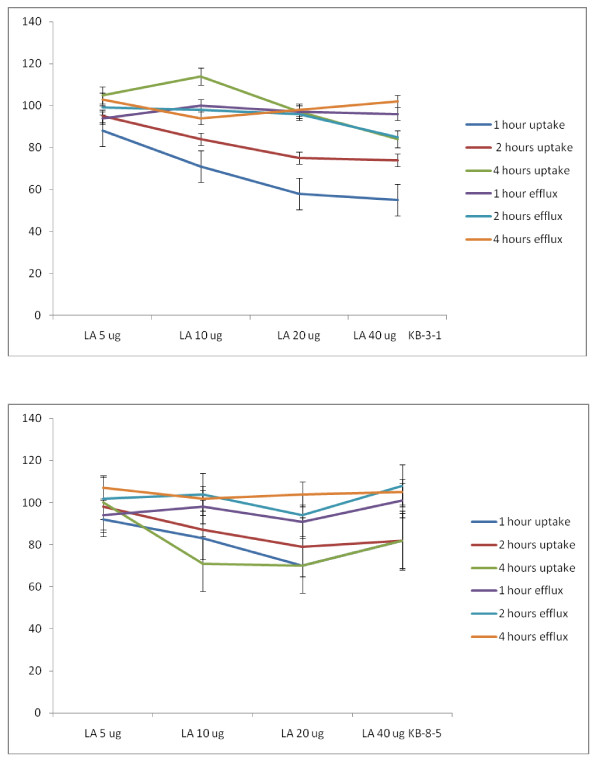
**Uptake and efflux of [^3^H] vincristine by vincristine-sensitive (KB-3-1) and vincristine-resistant (KB-Ch^R^-8-5) tumor cells *in vitro *in the presence of LA**. All values are shown as % of the control.

In the presence of various n-6 fatty acids such as LA, GLA, DGLA and AA the uptake and efflux of radiolabeled vincristine was substantially altered. When KB-3-1 cells were incubated for different time intervals with LA there was no significant change in the uptake and efflux of vincristine as shown in Figure 17. Both uptake and efflux of vincristine by KB-3-1 cells were almost similar that resulted in no significant increase in accumulation of vincristine in the cells. This suggests that LA is unlikely to enhance the cytotoxic action of vincristine when both are added together to the tumor cells. In a similar fashion, LA did not substantially enhance the accumulation of vincristine in drug-resistant cells KB-Ch^R^-8-5 cells since both the uptake and efflux of vincristine were almost similar (see Figure 17).

##### Studies with GLA

**Figure 18:**
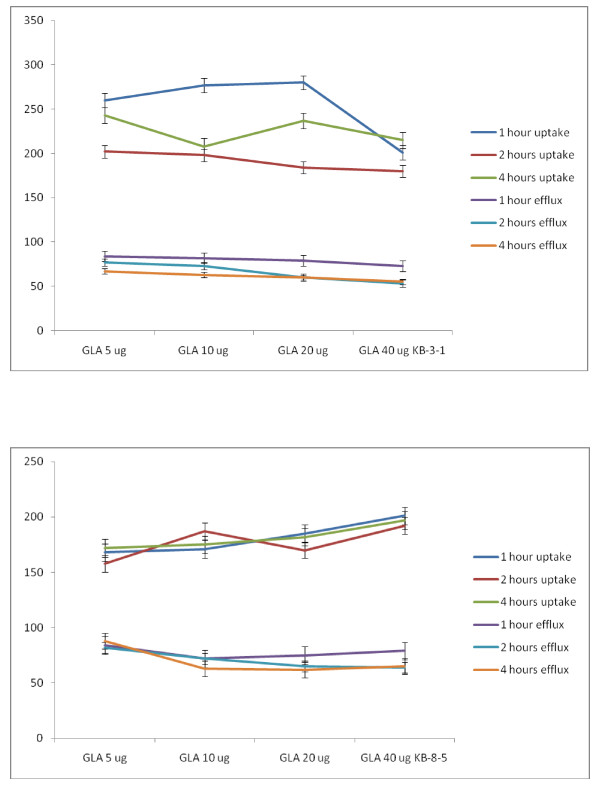
**Uptake and efflux of [^3^H] vincristine by vincristine-resistant (KB-Ch^R^-8-5) tumor cells *in vitro *in the presence of GLA**. All values are expressed as % of control.

Unlike LA, GLA was effective in increasing the uptake of vincristine by 1.5 to 2-fold in both KB-31 and KB-Ch^R^-8-5 cells as shown in Figure 18.

##### Studies with DGLA

**Figure 19:**
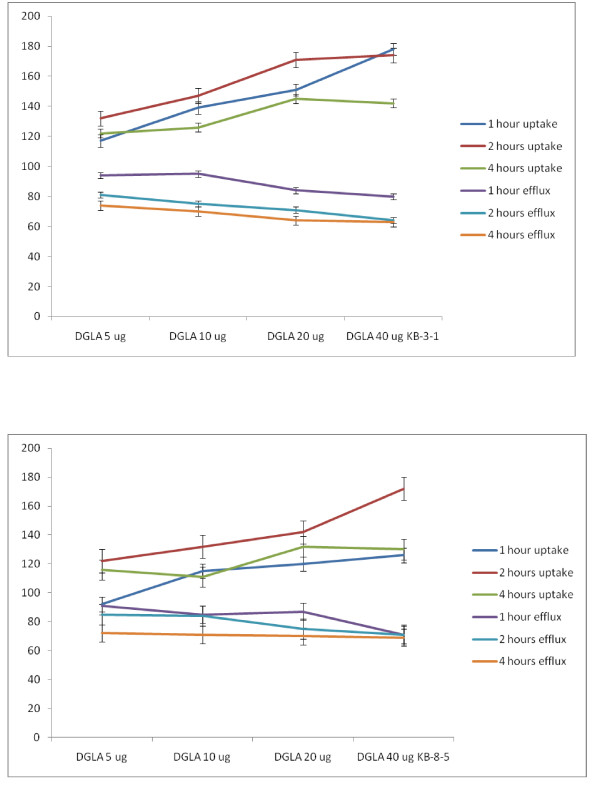
**Uptake and efflux of [^3^H] vincristine by vincristine-sensitive (KB-3-1) tumor cells in vitro in the presence of DGLA**. All values are expressed as % of control.

Similar to GLA, DGLA was also effective in enhancing the uptake of vincristine and reducing the efflux resulting in enhanced intracellular concentration of the anti-cancer drug. But DGLA was definitely much less effective compared to GLA. The results of the studies done with DGLA are given in Figures 19. It may be noted that GLA is much more effective against KB-Ch^R^-8-5 (vincristine-resistant cells) compared to DGLA in increasing the uptake of vincristine. Thus, GLA is more effective than DGLA both in enhancing the uptake of vincristine in KB-Ch^R^-8-5 cells and reducing its efflux. This suggests that GLA may be more effective in sensitizing the vincristine-resistant cells to the cytotoxic action of vincristine compared to DGLA. Since both GLA and DGLA contain 3 double bonds (GLA = 18:3 while DGLA is 20:3), it appears as though the number of double bonds may not be responsible for this property (namely, uptake and efflux of vincristine by KB-Ch^R^-8-5 cells) shown by the cells. It could be attributed to the number of carbon atoms.

##### Studies with AA

**Figure 20:**
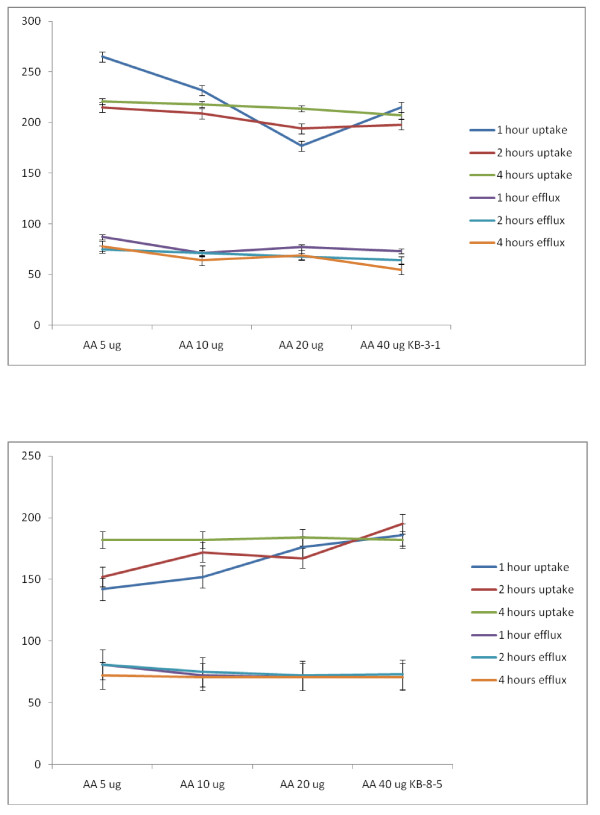
**Uptake and efflux of [^3^H] vincristine by vincristine-sensitive (KB-3-1) tumor cells in vitro in the presence of AA**. All values are expressed as % of control.

It is seen from the results shown in Figure 20 that AA is more effective than GLA and DGLA in enhancing the uptake and decreasing the efflux of vincristine by both the vincristine-sensitive (KB-3-1) and vincristine-resistant (KB-Ch^R^-8-5) cells. Since AA is more unsaturated and contain 20 carbon atoms (AA = 20:4), it appears as though both the chain length and unsaturation may contribute to the ability of the AA in enhancing the vincristine drug uptake and decrease in its efflux (vincristine) by tumor cells that could result in higher intracellular concentration(s) of the anti-cancer drug in drug-sensitive and drug-resistant cancer cells.

#### Effect of various n-3 fatty acids

##### Studies with ALA

**Figure 21:**
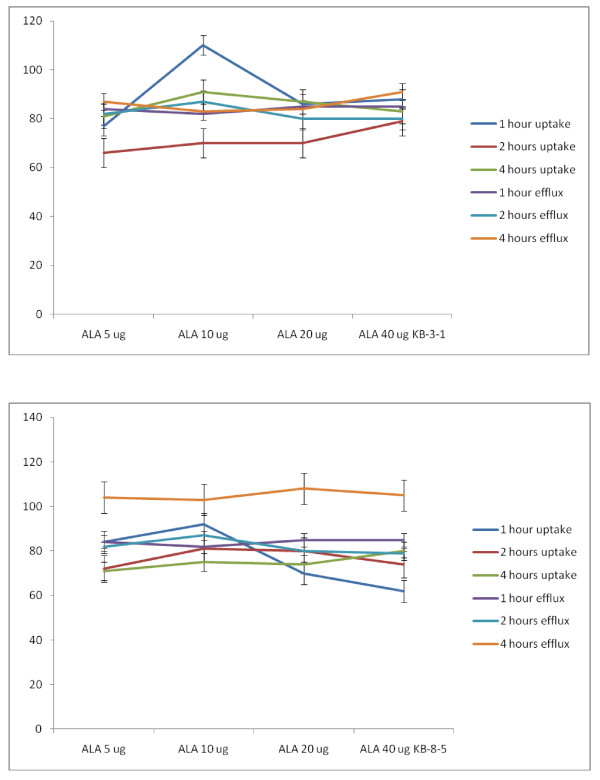
**Uptake and efflux of [^3^H] vincristine by vincristine-sensitive (KB-3-1) and vincristine-resistant (KB-Ch^R^-8-5) tumor cells *in vitro *in the presence of ALA**. All values are % of control.

**Figure 22:**
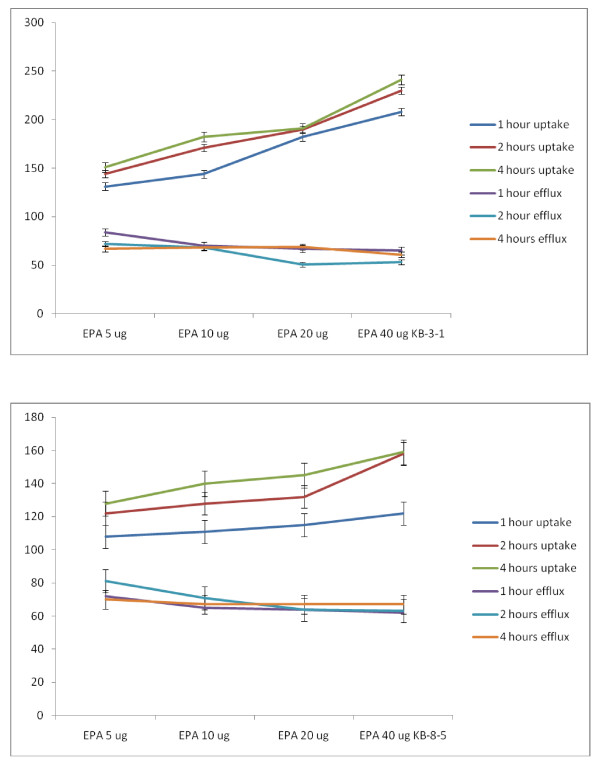
**Uptake and efflux of [^3^H] vincristine by vincristine-sensitive (KB-3-1) vincristine-resistant (KB-Ch^R^-8-5) tumor cells *in vitro *in the presence of EPA**. All values are % of control.

**Figure 23:**
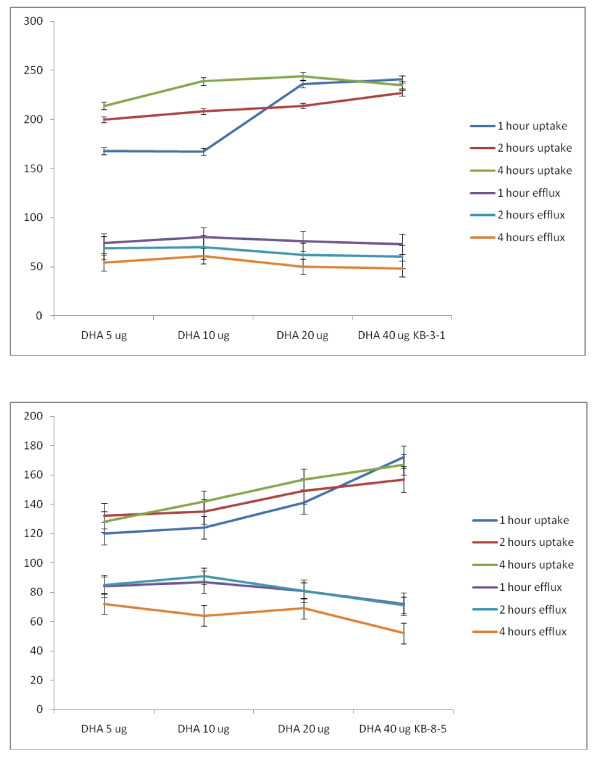
**Uptake and efflux of [^3^H] vincristine by vincristine-sensitive (KB-3-1) vincristine-resistant (KB-Ch^R^-8-5) tumor cells *in vitro *in the presence of DHA**. All values are % of control.

Similar studies were also performed with n-3 fatty acids: ALA, EPA and DHA. It is evident from these results shown in Figures 21, 22 and 23 that similar to LA, ALA is not effective in enhancing the uptake and decreasing the efflux of vincristine by KB-3-1 and KB-Ch^R^-8-5 cells; whereas EPA (n-3, 20:5) and DHA (n-3, 22:6) significantly enhanced uptake and decreased efflux of vincristine by KB-3-1 and KB-Ch^R^-8-5 cells such that the intracellular concentrations of the anti-cancer drug is increased leading to death of the tumor cells. Of all the three n-3 fatty acids tested DHA is the most effective followed by EPA while ALA is the least effective or has no effect (DHA > EPA > ALA). In addition both n-6 and n-3 fatty acids were more effective at higher concentrations (say 20 and 40 μg) while they were less effective at lower doses (5-10 μg). In general, GLA, AA, EPA and DHA were more effective in enhancing the uptake of vincristine but had a small effect in decreasing the efflux of the drug both in the KB-3-1 and KB-Ch^R^-8-5 cells. This suggests that GLA, AA, EPA and DHA are more effective in enhancing the uptake of the anti-cancer drugs and had less effect in decreasing the efflux of the drugs. Nevertheless, GLA, AA, EPA and DHA effectively increased intracellular concentration of vincristine, an action that seems to be responsible for the augmentation of the cytotoxic action of anti-cancer drugs by PUFAs.

### Effect of combined action of sub-optimal concentrations of vincristine and sub-optimal doses of PUFAs on KB-Ch^R^-8-5 cells *in vitro*

If it is true that fatty acids are able to enhance the uptake and decrease the efflux of anti-cancer drugs, then it is anticipated that a combination of sub-optimal doses of anti-cancer drugs and sub-optimal doses of fatty acids could induce substantial cytotoxic action on tumor cells. Hence, studies were performed on the cytotoxic action of a combination of sub-optimal doses of vincristine and sub-optimal doses of fatty acids on their cytotoxic action on vincristine-resistant (KB-Ch^R^-8-5) cells *in vitro*.

**Figure 24:**
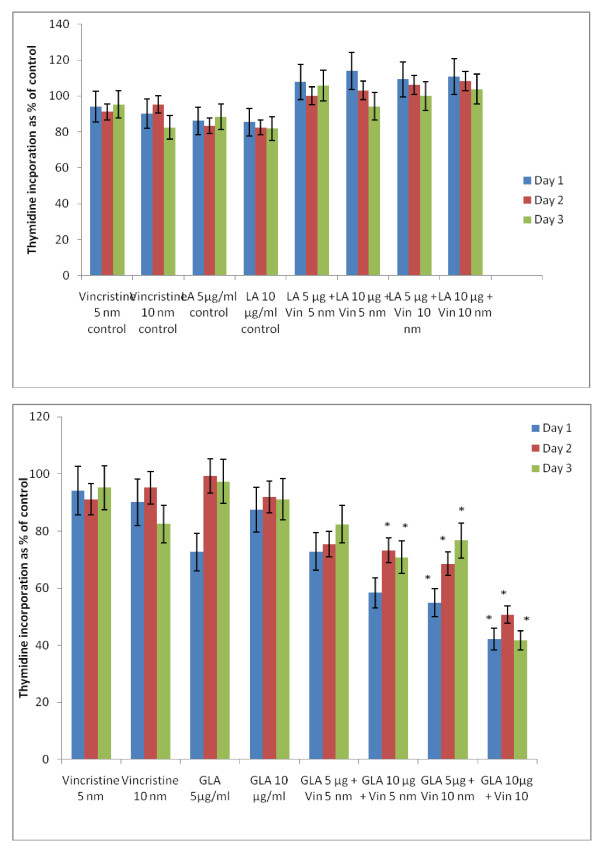
**Effect of sub-optimal doses of LA and GLA and vincristine on the proliferation of vincristine-resistant (KB-Ch^R^-8-5) tumor cells in vitro**. All values are mean ± SE. In the study with sub-optimal doses of GLA, *P < 0.05 compared to control (vincristine 5 nm and 10 nm and GLA 5 μg/ml and 10 μg/ml).

**Figure 25:**
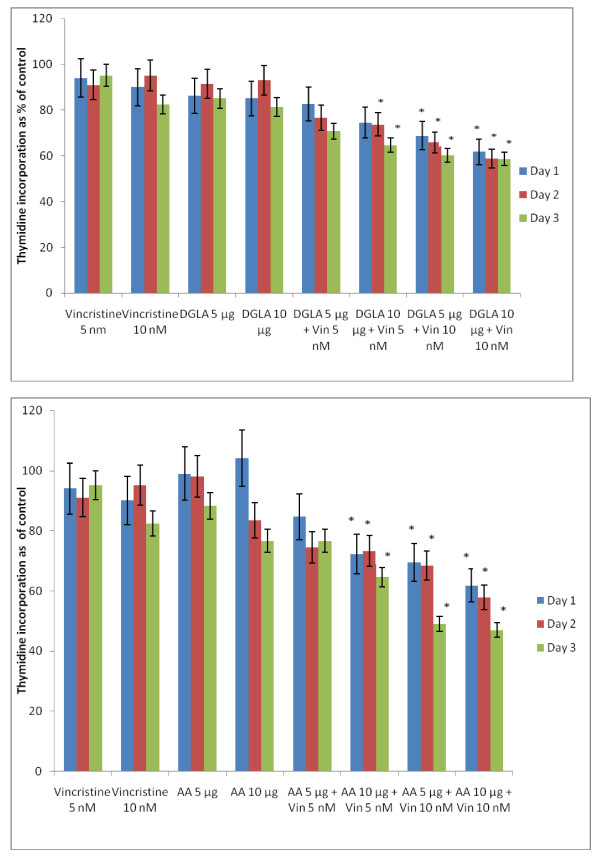
**Effect of sub-optimal doses of DGLA and AA and vincristine on the proliferation of vincristine-resistant (KB-Ch^R^-8-5) tumor cells in vitro**. All values are expressed as Mean ± SE. *P < 0.05 compared to control (vincristine and DGLA and AA alone controls).

**Figure 26:**
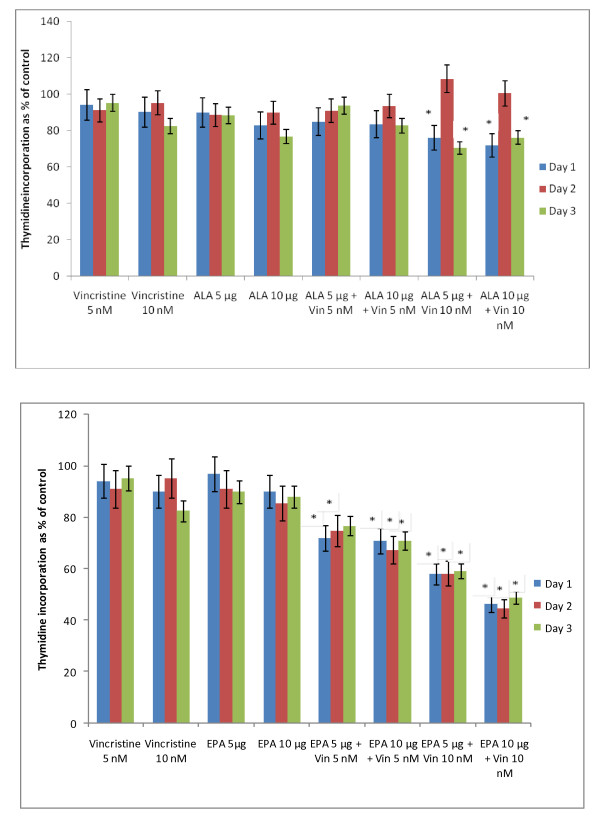
**Effect of sub-optimal doses of ALA, EPA and vincristine on the proliferation of vincristine-resistant (KB-Ch^R^-8-5) tumor cells in vitro**. All values are mean ± SE. *P < 0.05 compared to control.

**Figure 27:**
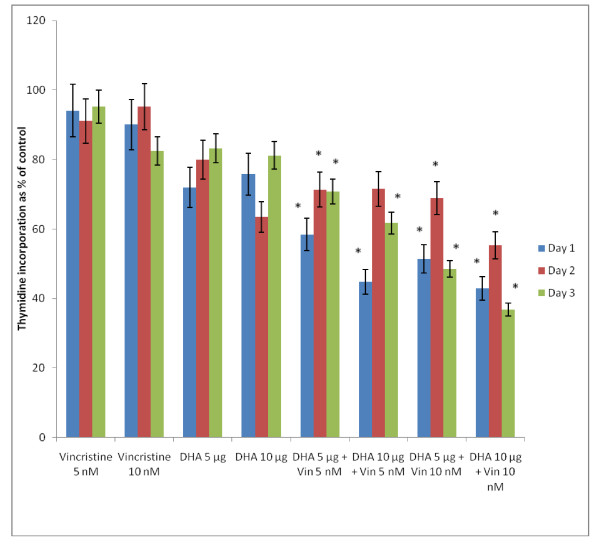
**Effect of sub-optimal doses of DHA and vincristine on the proliferation of vincristine-resistant (KB-Ch^R^-8-5) tumor cells in vitro**. P < 0.05 compared to control.

**Figure 28:**
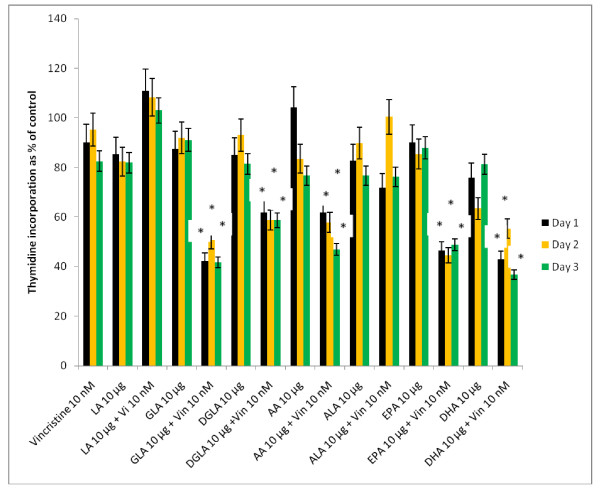
**Effect of sub-optimal doses of n-6 and n-3 fatty acids (10 μg of fatty acids) and vincristine (10 nm) on the proliferation of vincristine-resistant (KB-Ch^R^-8-5) tumor cells *in vitro***. * P < 0.05 compared to control.

In this study, KB-Ch^R^-8-5 (vincristine-resistant tumor) cells were exposed to sub-optimal doses of vincristine and sub-optimal doses of various n-6 and n-3 PUFAs. The results of these studies given in Figures 24, 25, 26, 27 and 28 showed that when KB-Ch^R^-8-5 cells were exposed to 5 nm and 10 nm of vincristine and 5 μg and 10 μg of various fatty acids did not show any substantial increase in the number of dead cells compared to the control. Thus, 5 nm and 10 nm of vincristine and 5 μg and 10 μg of fatty acids when used alone could be considered as sub-optimal doses at which there is no increase in the number of dead cells in comparison to control.

On the other hand, when KB-Ch^R^-8-5 cells were exposed to a combination of sub-optimal doses of vincristine and various n-6 and n-3 fatty acids, a significant increase in the number of dead cells was observed. Of all the fatty acids tested, GLA, DGLA, AA, EPA and DHA were found to be the most effective in enhancing the cytotoxicity of sub-optimal doses of vincristine. LA was found to the least effective whereas ALA was effective only to a limited extent in enhancing the cytotoxicity of sub-optimal doses of vincristine. These results suggest that a combination of sub-optimal doses of vincristine and GLA, DGLA, AA, EPA and DHA fatty acids are effective in substantially enhancing the death of vincristine-resistant (KB-Ch^R^-8-5) tumor cells *in vitro*.

For easy understanding, a summary of the results obtained with sub-optimal doses of n-6 and n-6 fatty acids (10 μg of fatty acids) and vincristine (10 nm) is given in Figure 28.

It is evident from the results shown in Figures 24, 25, 26, 27 and 28 that there is a gradual enhancement in the cytotoxic action of sub-optimal doses of a combination of vincristine and fatty acids as given below:

Fatty acid 5 μg + Vincristine 5 nm < Fatty acid 10 μg + Vincristine 5 nm < Fatty acid 5 μg + Vincristine 10 nm < Fatty acid 10 μg + Vincristine 10 nm.

Thus, the most effective sub-optimal doses of vincristine and fatty acid in inducing the death of vincristine-resistant cells is vincristine 10 nm + fatty acid 10 μg. Of all the fatty acids tested, the most effective fatty acid is DHA. With regard to the fatty acids, the effectiveness of the fatty acids when used at sub-optimal doses in combination with vincristine is as follows: DHA > GLA > AA = EPA.

### Uptake and efflux of fatty acids by vincristine-sensitive and vincristine-resistant cells in vitro

It is evident from the results shown in Figures 16, 17, 18, 19, 20, 21, 22 and 23 that the uptake and efflux of vincristine by vincristine-sensitive (KB-3-1) and vincristine-resistant (KB-Ch^R^-8-5) cells is modified by various n-6 and n-3 fatty acids. GLA, AA, EPA and DHA enhanced the uptake of vincristine and reduced its efflux in these two cell lines. As a result, the intracellular concentration of vincristine is enhanced resulting in apoptosis of tumor cells. Thus, GLA, AA, EPA and DHA are capable of augmenting the apoptosis of both vincristine-sensitive and vincristine-resistant cells to vincristine. But, no data is available as to the uptake and efflux of fatty acids themselves in KB-3-1 and KB-Ch^R^-8-5 cells. Hence, we studied the uptake and efflux of fatty acids in these two cell lines.

**Figure 29:**
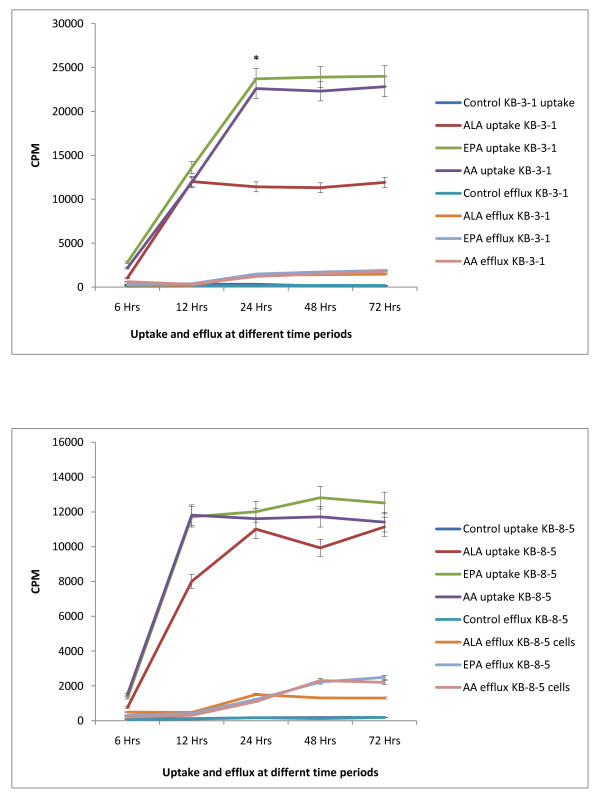
**Uptake and efflux of labeled fatty acids (ALA, AA, EPA) by vincristine-sensitive (KB-3-1) vincristine-resistant (KB-Ch^R^-8-5) cells *in vitro *at different time intervals**.

**Figure 30:**
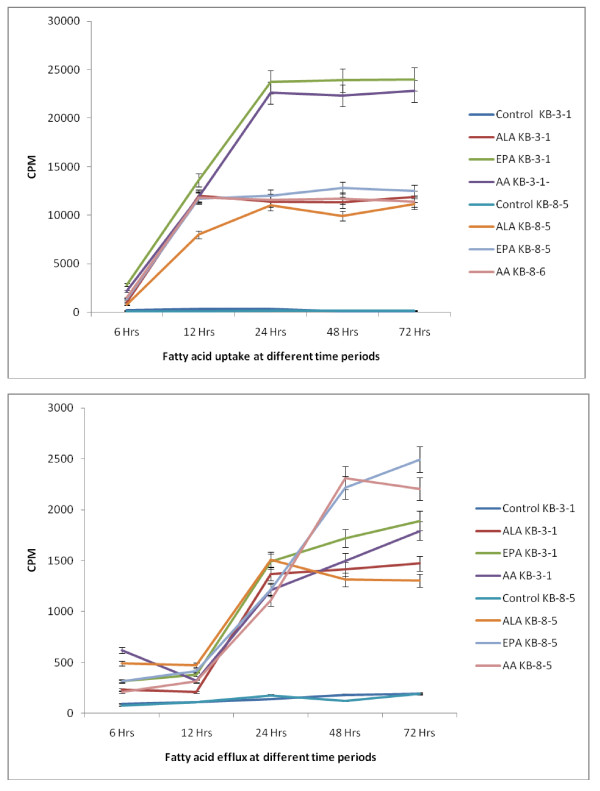
**A comparison of the uptake and efflux of fatty acids (ALA, AA, EPA) by vincristine-sensitive (KB-3-1) vincristine-resistant (KB-Ch^R^-8-5) cells *in vitro *at different time intervals**.

The results of this study given in Figures 29 and 30, suggest that the uptake of fatty acids is higher and the efflux is lower in KB-3-1 cells compared to the uptake and efflux of the same fatty acids in KB-Ch^R^-8-5 cells. This could be one reason for the higher sensitivity of KB-3-1 cells to the cytotoxic action of fatty acids compared to KB-Ch^R^-8-5 cells. Of the three fatty acids tested, the uptake and efflux of DHA = AA > ALA both in KB-3-1 and KB-Ch^R^-8-5 cells.

## Discussion

**Figure 31:**
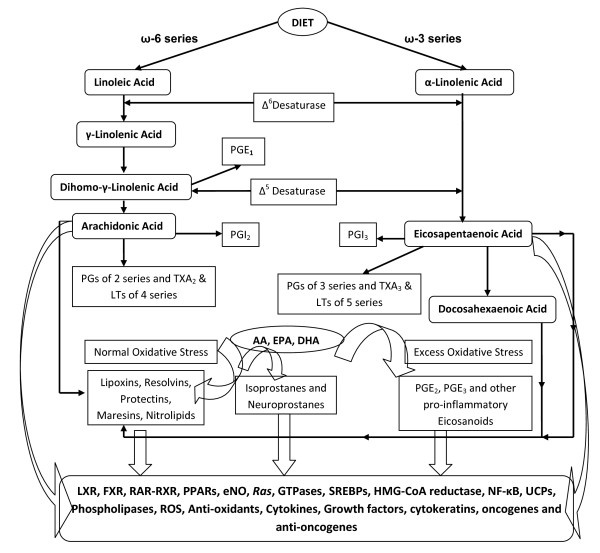
**Metabolism of essential fatty acids**.

Previous studies showed that PUFAs such as GLA, AA, EPA and DHA have tumoricidal action (1-6, [31-36], for metabolism of essential fatty acids, EFAs, and see Figure 31). This tumoricidal action of fatty acids seems to depend on their ability to augment free radical generation and lipid peroxidation process in the tumour cells [1,4,5,13,25]. The involvement of free radicals is supported by the observation that antioxidants such as vitamin E, superoxide dismutase (SOD) and to some extent BHA and BHT (butylated hydroxy anisole and butylated hydroxytoluene, respectively) can prevent the tumoricidal action of PUFAs [1, 4, 13 and the present study]. Both free radicals and lipid peroxides induce damage to a variety of enzymes, proteins and DNA and thus, lead to cell death [37]. Further free radicals deplete ATP levels in the cells and cause apoptosis [38-40]. Since PUFAs enhance free radical generation and lipid peroxidation process and thus, induce apoptosis, it suggests that these events lead to depletion of ATP levels in the tumor cells which leads to their death. This implies that an interaction exists among fatty acids, lipid peroxidation process, apoptosis and genes/oncogenes that regulate apoptotic process [12].

In this context, it is important to note that reactive oxygen metabolite hydrogen peroxide (H_2_O_2_) stimulates AA release and thromboxane A_2 _(TXA_2_) synthesis in the rat alveolar macrophage, but does not stimulate 5-lipoxygenase metabolism to form leukotriene B_4 _(LTB_4_), LTC_4_, or 5-hydroxyeicosatetraenoic acid (5-HETE). H_2_O_2 _dose-dependently inhibited synthesis of LTB_4_, LTC_4_, and 5-HETE induced by the agonists A23187 (10 microM) and zymosan (100 micrograms/ml), over the same concentration range at which it augmented synthesis of the cyclooxygenase products TXA_2 _and 12-hydroxy-5,8,10-heptadecatrienoic acid. This action of H_2_O_2 _on 5-lipoxygenase and cyclo-oxygenase synthesis is due to the ability of H_2 _O_2 _to deplete cellular ATP, a cofactor for 5-lipoxygenase. Thus, H_2_O_2 _can act both as an agonist for macrophage AA metabolism, and as a selective inhibitor of the 5-lipoxygenase pathway by its ability to deplete ATP [40]. These results are interesting in the light of the observation that LTs enhance the growth of tumor cells [41,42]. This can be interpreted to mean that free radicals, especially H_2_O_2_, induce apoptosis of tumor cells by depleting the cells (a) of their ATP content, and (b) of LTs that are tumor growth promoters by selectively inhibiting 5-lipoxygenase activity [43,44]. But, this growth inhibitory action of LTs is not without controversy since some studies did suggest that LTB_4 _and LTC_4 _may have tumor growth inhibitory actions [45,46]. These controversial results could be due to the differences tumor cell lines studied and the doses of LTs employed.

In the light of these evidences, the result of the present study wherein it is noted that GLA, AA, EPA and DHA are cytotoxic to both vincristine-sensitive and resistant tumor cells is interesting. The inability of both indomethacin and NDGA, a CO and LO inhibitors respectively, to block, while the ability of vitamin E and SOD to completely inhibit the cytotoxic action of GLA and DHA on KB-3-1 cells suggested that in all probability PGs, LTs and TXs do not participate in the cytotoxic action of PUFAs. It is possible that KB-3-1 cells and possibly. KB-Ch^R^-8-5 cells do not form significant amounts of LTs that are known to enhance the growth of tumor cells [41,42]. The failure of calmodulin antagonists: chlorpromazine (CPZ) and trifluoperazine (TFP) to block the cytotoxic action of PUFAs indicates that calmodulin does not play a role in the proliferation of KB-3-1 and KB-Ch^R^-8-5 cells. Though antioxidant vitamin E completely blocked the cytotoxic action of PUFAs (GLA, AA, EPA and DHA), failure of BHA and BHT, synthetic antioxidants, to show similar inhibitory action is rather surprising. This may mean that the free radicals that are scavenged by vitamin E and BHA and BHT are different and/or act on the free radical-mediated cellular processes in vastly different manner(s). Both mannitol and catalase were partially effective in inhibiting the GLA- and DHA-induced cytotoxicity (Figure 8) suggesting that there is only partial involvement of hydroxyl radical and H_2_O_2 _respectively in their cytotoxic action [47]. On the other hand, vitamin E completely blocked the cytotoxic actions of GLA, AA, EPA and DHA on both vincristine-sensitive and resistant cells by suppressing free radical generation in these cells (Figure 15). The ability of vitamin E but not of synthetic antioxidants BHA and BHT and only partial inhibition by catalase and mannitol suggests that, perhaps, all types of free radicals (superoxide anion, H_2_O_2_, hydroxyl radicals) and lipid peroxides play a role in the induction of apoptosis of tumor cells by PUFAs. The potent action of vitamin E in the inhibition of PUFA-induced tumoricidal action could also be attributed to its lipid soluble nature, its ability to protect glutathione against microsomal lipid peroxidation [48] and block lipid peroxidation chain reaction [49]. As a result of these actions, vitamin E is able to remove free radical intermediates and prevent the oxidation reaction from continuing. This is supported by the observation that vitamin E prevented PUFA-induced free radical generation and formation of lipid peroxides in vincristine-sensitive cells (Figure 15).

Since drug-resistance is a major issue in clinical practice, we studied whether PUFAs have the ability to alter the sensitivity of vincristine-resistant cells to the cytotoxic action of vincristine *in vitro*. Based on the results obtained in the present study, it is clear that certain PUFAs are not only capable of selectively killing the tumor cells with little effect on normal cells at the concentrations tested but are also capable of enhancing the uptake of anti-cancer drugs both by drug-sensitive and drug-resistant tumor cells (see Figures 17, 18, 19, 20, 21, 22 and 23) and may reverse tumor cell drug resistance *in vitro*. Thus, GLA, AA, EPA and DHA are able to bring about their tumoricidal action (i) by enhancing free radical generation and lipid peroxidation process in tumor cells and (ii) by increasing intracellular concentration of the anti-cancer drugs.

These results are further supported by the observation that when sub-optimal doses of vincristine and PUFAs are used, the cytotoxic action of vincristine was substantially enhanced especially by GLA, DGLA, AA, EPA and DHA (Figures 24, 25, 26, 27 and 28). In addition, uptake of PUFAs is higher in vincristine-sensitive cells compared to vincristine-resistance cells while the efflux is higher in the vincristine-resistant cells compared with vincristine-sensitive cells (Figures 29 and 30). These results indicate that, in general, drug-resistant cells show higher efflux compared to drug-sensitive tumor cells not only to anti-cancer drugs but also to PUFAs whereas drug-sensitive cells show higher uptake and decreased efflux to anti-cancer drugs and PUFAs (Figures 17, 18, 19, 20, 21, 22 and 23 and Figures 29 and 30). These studies imply that PUFAs could be used to reduce drug-resistance or reverse drug-resistance by various tumor cells such that chemotherapeutic drugs could bring about their tumoricidal action more effectively.

Previous studies [1-5,11,14] suggested that PUFAs (if not all at least GLA, AA, EPA and DHA) have differential toxicity towards normal and tumor cells indicating that normal and tumor cells metabolize fatty acids differentially. For instance, AA is metabolized to produce the 5-lipoxygenase metabolite, 5-HETE (5-hydroxyeicosatetraenoic acid) by prostate cancer cells that stimulated their growth, suggesting that 5-HETE is a survival factor for these cells. Prostate cancer cells constitutively produce 5-HETE and exogenous arachidonate markedly increases the production of 5-HETE, while inhibition of 5-lipoxygenase induced apoptosis in both hormone-responsive (LNCaP) and -nonresponsive (PC3) human prostate cancer cells. Apoptosis was specific for 5-lipoxygenase-programmed cell death since it was not observed with inhibitors of 12-lipoxygenase, cyclooxygenase, or cytochrome P450 pathways of AA metabolism. Exogenous 5-HETE protected these cells from apoptosis induced by 5-lipoxygenase inhibitors, confirming a critical role of 5-lipoxygenase activity in the survival of these cells [50]. Hence, it can be said that the way free fatty acids are metabolized by tumor cells, be drug-sensitive and drug-resistant cells, influence survival and progression of cancer. For example, free AA and GLA are tumoricidal but when AA is converted to form 5-HETE by 5-lipoxygenase, the tumor cells are stimulated to grow [51,52].

Cyclooxygenase-2 (COX-2) is up-regulated in many cancers that may explain as to why COX-2 inhibitors prevent colon cancer. This, in part, could be attributed to an accumulation of the substrate (AA) or diversion of the substrate into another pathway. For example, colon adenocarcinomas overexpress AA-utilizing enzyme, fatty acid-CoA ligase (FACL) 4, in addition to COX-2. Thus, unesterified arachidonic acid in cells is a signal for induction of apoptosis. Tumor cells engineered with inducible overexpression of COX-2 and FACL4 act as "sinks" for unesterified AA as evidenced by the observation that activation of the enzymatic sinks blocked apoptosis, and the reduction of cell death was inversely correlated with the cellular level of AA. Cell death caused by TNF-α is prevented by removal of unesterified AA, suggesting that cellular level of unesterified AA and other unsaturated fatty acids is a general mechanism by which apoptosis is regulated and that COX-2 and FACL4 promote carcinogenesis by lowering this level [53-56]. Furthermore, NSAIDs up-regulated 15-LOX-1 and 15-LOX-1 inhibition blocked NSAID-induced apoptosis, which was restored by 13-S-HODE (13-S-hydroxyoctadecadienoic acid, is the product of 15-LOX-1 protein, the other product of 15-LOX-1 is 15-S-HETE, but in this study 15-S-HETE formation was not noted) but not by its parent, LA. Thus, NSAIDs induce apoptosis in colon cancer cells via up-regulation of 15-LOX-1 in the absence of COX-2 [57-59]. Hydroperoxides generated by 5-, 12-, or 15-lipoxygenases from linoleate, linolenate, or arachidonate (hydroperoxides may be detected as lipid peroxides by MDA reaction as was done in the present study), and the corresponding hydroxides induced apoptosis of erythroleukemia and neuroblastoma cells in a concentration- and time-dependent manner, while the terminal products of the arachidonate cascade (i.e., leukotrienes, prostaglandins and thromboxanes) were not cytotoxic [60]. These results are supported by the results of the present study wherein it is noted that both CO and LO inhibitors did not block the cytotoxic action of PUFAs. Thus, free unsaturated fatty acids need to be converted to their respective hydroxides to bring about their tumoricidal action. In addition, many AA metabolites serve as growth signaling molecules. 5-lipoxygenase (5-LO) pathway metabolite 5(S)-hydrooxyeicosa-6E,8C,11Z,14Z-tetraenoic acid (5-HETE) has a growth stimulatory action on breast cancer cells whereas selective reduction in the levels of 5-HETE but not cyclooxygenase inhibitors reduced growth, increased apoptosis, down-regulated bcl-2, up-regulated bax, and increased G1 arrest. 5-LO inhibition up-regulated peroxisome proliferator-activated receptor-α (PPAR-α) and PPAR-γ expression, and were growth inhibited when exposed to relevant PPAR agonists. These results suggest that disruption of the 5-LO signaling pathway mediates growth arrest and apoptosis in breast cancer cells, partly, by the induction of PPARs and activation of PPARs with shunted endoperoxides [61,62]. These results imply that delivery of free unsaturated fatty acids to the tumor cells and generation of hydroperoxides by 5- 12-, or 15-lipoxygenases from various PUFAs such as AA, and simultaneous inhibition of COX-2 enzyme could lead to apoptosis of tumor cells. In addition, PUFAs suppress fatty acid synthase enzyme and thus, induce apoptosis of tumor cells [63-69].

**Figure 32:**
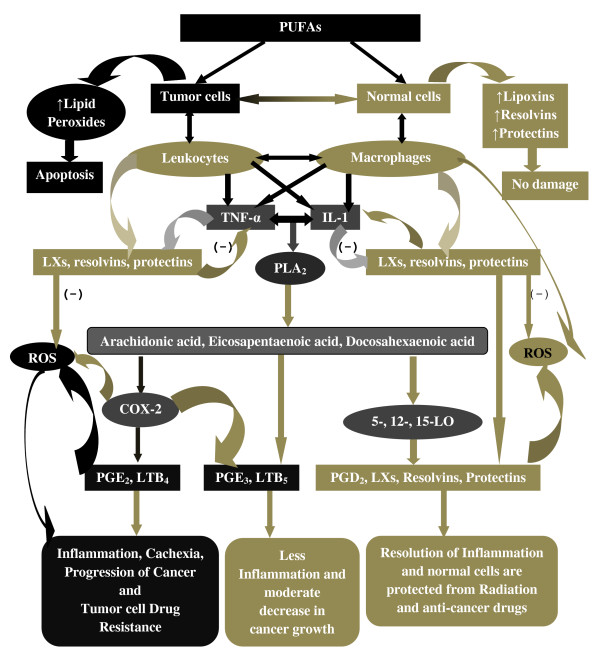
**Scheme showing the differential metabolism of PUFAs in normal and tumor cells**. Normal cells exposed to anti-cancer drugs and radiation produce increased amounts of ROS. In response to this, normal cells produce enhanced amounts of lipoxins, resolvins and protectins from PUFAs that are released by the activation of phospholipase A_2_. Infiltrating or local leukocytes and macrophages produce enhanced amounts of IL-6 and TNF-α that, in turn, enhance ROS generation and cause inflammation. If cell stores of PUFAs are adequate, activation of phospholipase A_2 _(the type of phospholipase activated in normal and tumor cells may be distinctly different) leads to release of PUFAs to be converted to lipoxins, resolvins and protectins, which suppress leukocyte and macrophage activation, ROS generation and inflammation to protect normal cells from apoptosis. In the case of tumor cells, infiltrating leukocytes and macrophages produce enhanced amounts of IL-6 and TNF-α that produce excess amounts of ROS leading to inflammation. ROS damage DNA and aid in the progression of cancer. Tumor cells have decreased amounts of PUFAs that get are converted to pro-inflammatory eicosanoids due to activation of COX-2 and LO and thus, inflammation and cancer growth are perpetuated. Normal cells supplemented with PUFAs produce adequate amounts of lipoxins, resolvins and protectins that protect them from ROS, suppress inflammation and prevent actions of mutagens and carcinogens. On the other hand, tumor cells supplemented with PUFAs generate more free radicals, show enhanced lipid peroxidation that lead to apoptosis. Thus, normal cells exposed to PUFAs produce cytoprotective lipoxins, resolvins and protectins while tumor cells generate toxic hydroperoxy fatty acids.

Based on the preceding discussion, one intriguing possibility is that PUFAs are differentially metabolized by normal and tumor cells and drug-sensitive and drug-resistant cells (in addition to the differential uptake and efflux shown by the drug resistant tumor cells). This is so because PUFAs form precursors to both cytotoxic and cytoprotective molecules. For instance, as discussed above, generation of hydroperoxides by 5- 12-, or 15-lipoxygenases from linoleate, linolenate, or AA have cytotoxic actions and induce apoptosis of tumor cells. In contrast, lipoxins, resolvins, and protectins (including neuroprotectin D_1_) have cytoprotective properties by virtue of their anti-inflammatory actions. Thus, it is likely that when tumor cells are exposed to adequate amounts of PUFAs cytotoxic metabolites are generated that induce apoptosis of tumor cells, whereas normal cells convert PUFAs to cytoprotective molecules such as lipoxins, resolvins and protectins [70-81]. This is supported by the observation that DHA is toxic to tumor cells but protects normal neural cells from stress-induced apoptosis and is less or not cytotoxic to normal cells [1,3,11]. DHA induces apoptosis of neuroblastoma cells due to its conversion to 17-hydroxydocosahexaenoic acid (17-HDHA) via 17-hydroperoxydocosahexaenoic acid (17-HpDHA) through 15-lipoxygenase and autoxidation [82] and as a result tumor cells do not produce (or form negligible amounts) of the anti-inflammatory lipid mediators such as resolvins and protectins. 17-HpDHA is cytotoxic to tumor cells [70] and DHA itself could inhibit secretion of PGE_2_. Thus, the cytotoxic action of DHA on neuroblastoma and other tumor cells could be due to the production of hydroperoxy fatty acids and restricted production of resolvins and protectins that are cytoprotective in nature [83-86]. In a similar fashion, it is possible that when normal cells are exposed to GLA, AA, and EPA significant amounts of lipoxins and resolvin and other similar compounds are formed, whereas tumor cells would accumulate respective, prostanoids, leukotrienes, thromboxanes and cyclopentanone prostaglandins and respective hydroperoxy fatty acids that are toxic. Hence, it is proposed that normal cells metabolize PUFAs to produce cytoprotective lipids such as lipoxins, resolvins and protectins while tumor cells generate toxic hydroperoxy fatty acids [87]. This differential metabolism of PUFAs by normal and tumor cells may explain why PUFAs are toxic to tumor but not to normal cells (see Figure 32). The fact that both CO and LO inhibitors did not inhibit the cytotoxic action of PUFAs while there is an increase in the formation of lipid peroxides in the tumor cells suggests that products other than PGs, LTs and TXs such as hydroperoxides (similar to 17-HpDHA) are formed that induce tumor cell death. In view of this, it would have been interesting had we measured various hydroperoxides, lipoxins, resolvins and protectins that are formed in tumor cells in the present study. In future, such studies need to be performed to confirm the proposals made above.

In summary, results of the present study and previous reports [1-17] indicate that

(i) free PUFAs and their hydroperoxides are toxic to tumor cells;

(ii) PUFAs- induced tumoricidal action could be attributed to their ability to enhance free radical generation and lipid peroxidation process;

(iii) PUFAs augment uptake and decrease efflux of anti-cancer drugs and thus, reverse tumor cell drug resistance;

(iv) Tumor cells have an effective efflux mechanism to overcome the cytotoxic action of anti-cancer drugs;

(v) CO and LO metabolites of PUFAs are less toxic to tumor cells compared to their (PUFAs) peroxides (measured in the present as total lipid peroxides) such as hydroperoxides (82);

(vi) Enhanced activity of CO and LO enzymes in tumor cells may serve as an escape mechanism to overcome tumoricidal action of free PUFAs;

(vii) A combination of PUFAs and anti-cancer drugs show enhanced cytotoxicity against tumor cells;

(viii) Normal cells may form enhanced amounts of cytoprotective molecules such as lipoxins, resolvins and protectins while tumor cells form cytotoxic lipid hydroperoxides and other peroxides (a concept that needs further confirmation); and

(ix) Tumor cell drug resistance could be due to increased formation of cytoprotective molecules such as lipoxins, resolvins and protectins, a concept that needs to be confirmed by further studies (see Figure 32).

## Competing interests

The authors declare that they have no competing interests.

## Authors' contributions

UND proposed the idea, designed experiments, interpreted the data and wrote the manuscript. NM did the experiments. All authors read and approved the final manuscript
